# Parental Knowledge of Breastfeeding and Nutrition: Influence on Oral Health and Self-Reported Early Childhood Caries in Preschool Children in Croatia

**DOI:** 10.3390/pediatric17020043

**Published:** 2025-04-03

**Authors:** Marija Badrov, Marija Matijević, Antonija Tadin

**Affiliations:** 1 Department of Restorative Dental Medicine and Endodontics, Study of Dental Medicine, School of Medicine, University of Split, 21000 Split, Croatia; marija.badrov@mefst.hr (M.B.); mb91904@mefst.hr (M.M.); 2 Department of Dental Medicine, University Hospital of Split, 21000 Split, Croatia

**Keywords:** oral health, breastfeeding, nutrition, dental caries, children, parents, knowledge

## Abstract

*Aim:* Parents’ knowledge of oral health plays a key role in shaping their children’s habits and preventing early childhood caries, particularly through breastfeeding and nutrition. This study aimed to assess parents’ knowledge of how dietary habits affect both oral health and early childhood caries rates. *Materials and methods:* An online cross-sectional survey was conducted among 595 parents of children aged 3 to 6 years old using a self-structured questionnaire. Sociodemographic data, the children’s characteristics, breastfeeding practices, daily diet, the perceived effects of diet on orofacial health, and self-reported dental caries were recorded. The data were analyzed using descriptive statistics, chi-square tests, and regression models. *Results:* One-third of the children had dental caries (200/595). The prevalence of caries was significantly higher among children from rural areas (40.5%) compared to those from urban areas (31.1%) (*p* = 0.021) and in low-income families (72.7%) compared to middle-income (35.4%) and high-income (25.1%) families (*p* = 0.002). Breastfeeding duration, bottle feeding, and night feeding were not significantly associated with the occurrence of caries. Only 11.1% of parents recognized the role of extended breastfeeding on a child’s demand over one year in promoting the development of tooth decay. Better knowledge was observed among parents with university degrees, in healthcare workers, and in parents with lower incomes (*p* < 0.05). *Conclusions:* Dental caries was prevalent, especially among children from rural areas and low-income families. Although there is no direct correlation between the duration of breastfeeding and dental caries, parental awareness of the preventive benefits of breastfeeding remains low. Education for parents about breastfeeding and nutrition’s impact on oral health can have a substantial effect on preventing ECC. Educational efforts aimed at specific audiences are necessary to boost knowledge and advance preventive strategies. This study must recognize its limitations due to its dependence on self-reported caries data. Subsequent research must include clinical dental evaluations to achieve findings that are both dependable and factual.

## 1. Introduction

Early childhood caries (ECC) stands as both a significant public health challenge and one of the most prevalent chronic diseases affecting children. The etiology of ECC is complex and includes dietary habits, the presence of cariogenic bacteria, oral hygiene practices, genetic predisposition, and socioeconomic factors that affect access to dental care and parental knowledge of oral health. If left untreated, ECC can affect quality of life and drive up healthcare costs, emphasizing the importance of effective prevention and treatment strategies [1,2]. The percentage of children affected by ECC shows extreme global variation from 12% to 98% based on different countries. The average rate of ECC throughout Europe stands at 36% but reaches its peak in Albania at 84.1% [3]. A 2001 study of preschool Croatian children aged 2 to 5 years showed an overall ECC prevalence of 30%, with 25% of female and 48% of male children affected [4]. Data from the Croatian Central Health Information System for the years 2013–2015 showed a DMFT index of 4.14 for 6-year-old children, with a range of 3.10 to 5.77 [5].

The health benefits of breastfeeding are far-reaching and include optimal nutrient absorption, strengthening the immune system, and facilitating proper orofacial structural development. Major health authorities, including the World Health Organization and the American Academy of Pediatrics, generally recommend exclusive breastfeeding for the first six months, followed by continued breastfeeding with complementary foods up to two years or beyond [6,7]. From an oral health perspective, breastfeeding promotes natural sucking behavior, which is essential for the proper development of the jaw and dental arch structures and can reduce the risk of malocclusion [8,9,10]. Most newborns can breastfeed, although there may be challenges due to a lack of maternal experience, nipple soreness, inadequate grip or posture, fatigue, and anatomical problems such as ankyloglossia. These factors can make breastfeeding difficult, which can result in poor nutrition and oral health and may lead to feeding problems [11]. The relationship between extended breastfeeding and early childhood caries (ECC) is a complex issue that remains only partially understood in the scientific community. Breastfeeding, in general, is considered protective of oral health development, as it supports the proper jaw and dental arch formation and provides important immune benefits. However, certain breastfeeding practices, particularly prolonged and frequent nighttime breastfeeding beyond the first year of life, may contribute to an increased risk of ECC, especially when accompanied by inadequate oral hygiene. Research suggests that extended nighttime breastfeeding exposes a child’s teeth to milk lactose for prolonged periods, creating a conducive environment for cariogenic bacteria to thrive. This is particularly problematic when oral hygiene practices are not properly implemented, as the milk residue left on the teeth provides a substrate for bacterial growth. This combination of prolonged milk exposure with poor plaque control may cause a predisposition to enamel demineralization and ultimately to the development of dental caries, especially in children due to the high vascular permeability to their teeth, making them more susceptible to bacterial colonization than adults. Therefore, although breastfeeding has many advantages, such beneficial practices should be balanced with adequate oral health practices and hygiene to avoid undesirable effects on oral tissue health [12,13,14].

Oral health and the prevention of dental caries are greatly influenced by food choices and dietary intake. The constant consumption of fermentable carbohydrates, especially sucrose, promotes the demineralization of tooth enamel through acid-forming bacterial activity. The risk of caries in children increases with the regular consumption of sugary snacks, sweetened drinks, and processed foods [15,16,17]. The transfer of harmful bacteria through using shared eating utensils or tasting foods can further increase the risks to dental health [18]. In contrast, a balanced diet rich in calcium, phosphorus, vitamin D, and other important nutrients supports enamel remineralization and overall oral well-being. Parental encouragement of healthy dietary patterns, including the regular consumption of fruit, vegetables, dairy products, meat and fish, contributes to robust enamel and reduces the potential for caries [19,20]. Given the lasting effects of early habits, it is crucial to educate parents about the role of nutrition in oral health to reduce the prevalence of ECC [16]. In studies of Croatian children, no significant association was found between dietary habits and oral health. These studies also indicated that dietary habits tend to worsen as children grow older [21]. Another study found that 50% of children aged 6 to 13 consume sweets, including cakes and cookies, with a higher frequency of consumption among boys than girls [22]. These findings suggest that while diet may have an impact on oral health, other factors may also play a role and that children’s eating habits may require more attention as they grow older.

Proper oral health practices are based on a solid foundation of oral health knowledge. Parents play a critical role in nurturing these habits, which include breastfeeding, dietary choices, and oral hygiene. Parents’ awareness of the correlation between their oral health and that of their children is crucial to preventing oral health issues in children. A study among pregnant women revealed that many women do not receive adequate dental care or information during pregnancy. This lack of education significantly affects their awareness of potential risks, such as the transmission of carious lesions to their child [23]. Misconceptions about nighttime breastfeeding, sugar consumption, and the timing of a first dental check-up can affect oral health. Increasing parental awareness is crucial to addressing these concerns [13,14,16,17,18,19]. A study in Croatia found that a significant proportion of pregnant women (49%) were unaware of how prolonged breastfeeding, bottle feeding, sleep feeding, and frequent feeding contribute to tooth decay and ECC, highlighting the importance of expectant mothers prioritizing the oral health of their offspring from the outset [24]. As there are few studies in Croatia investigating the relationship between breastfeeding, daily dietary habits, and the prevalence of ECC, this study aimed to assess parents’ knowledge of the effects of breastfeeding and diet on their children’s orofacial health. It also aimed to investigate how dietary habits and breastfeeding practices influence the incidence of early childhood caries. In addition, an attempt was made to identify correlations between the sociodemographic characteristics of the parents and their knowledge of these topics.

## 2. Materials and Methods

### 2.1. The Study Design and Data Collection

This descriptive cross-sectional study was conducted via a self-administered online survey that took place between 15 October and 30 November 2024. This study was conducted by the Department of Restorative Dentistry and Endodontics at the Faculty of Medicine, the University of Split. This study followed the guidelines of the Checklist for Reporting Results of Internet E-Surveys (CHERRIES) [25].

The survey was distributed anonymously via the Google Forms platform and shared via various online platforms, especially Facebook groups, to avoid selection bias and ensure a geographically diverse sample from different parts of Croatia. A combination of random sampling and snowballing was used, which is particularly well suited to an online environment and allows for convenient and accessible data collection from participants. Respondents were encouraged to invite other eligible individuals to participate, thereby increasing the reach of the survey. Participation was open to anyone who accessed the survey link, and the process ensured voluntary and anonymous participation. Informed consent was obtained at the beginning, with the participants receiving detailed information about the study on the first page of the questionnaire. By completing the questionnaire, participants gave their consent to take part in this study. No financial or other incentives were offered for their participation. Ethical approval of this study was granted by the Research Ethics Committee of the School of Medicine of the University of Split on 7 June 2024 (approval number 2181-198-03-04-24-0066).

### 2.2. The Participants and Sample Size

The inclusion criteria for this study were parents of preschool children aged 3 to 6 years from Croatia, while the exclusion criteria included parents of children with developmental disabilities and those whose children were either under 3 years or older than 6 years. The sample size was calculated for a prevalence study, aiming for a 95% confidence interval with a 5% margin of error. Based on these parameters, the required sample size was estimated at 385 participants. To take possible exclusions into account, a slightly larger sample size was aimed for. The sample size was calculated using the Sample Size Calculator (Inc. RaoSoft^®^, Seattle, WA, USA) based on the estimated number of children aged 3 to 6 years in Croatia (approximately 130,000) [26] and the reported number of births (n = 32,000) from the last census of 2023.

### 2.3. The Survey

The semi-structured questionnaire used in this study was developed in Croatia by the research team, which included specialists in endodontics and restorative dentistry, general dentists, and dental students. The questionnaire was based on the existing literature to achieve the specific objectives of the survey [9,10,13,14,16,17,18,19,24]. Following the development of the survey, a pilot test was conducted with a sample of ten suitable respondents. These pilot testers were asked to evaluate the clarity, usability, and technical functionality of the online survey. Their responses were not included in the final study sample, as they provided valuable feedback and suggestions that led to revisions of the survey. Additionally, the questionnaire’s reliability was evaluated, yielding a Cronbach’s alpha of 0.716 for items assessing knowledge of breastfeeding, nutrition, and oral health.

The final version of the questionnaire consisted of five sections and contained a total of 46 questions. The questionnaire took about 12 to 15 min to complete. The first section contained eight questions on the sociodemographic characteristics of the participants (e.g., their gender, age, education level, employment status, healthcare profession, household income, place of residence, and number of children). The second section consisted of five questions about their youngest child, including their child’s gender, age, weight (in kilograms), and height (in centimeters) for calculating BMI and the child’s dental caries status (whether the child had dental caries). The third section contained six questions about breastfeeding and the child’s feeding habits, e.g., the duration of breastfeeding, bottle feeding, sharing food with the child, the frequency of night feeds, the most common drink given to the child, and the timing of the introduction of solid foods. The fourth section comprised 15 dichotomous (“Yes/No”) questions about the child’s daily diet. The final section of the questionnaire assessed the respondents’ knowledge of the effects of breastfeeding and feeding on oral and facial health. This section comprised 12 questions with the answer options “Yes”, “No”, and “I don’t know”. The respondents received one point for each correct answer (“Yes”) and zero points for incorrect answers (“No/I don’t know”). The maximum score a respondent could achieve in this section was 12 points, indicating the level of knowledge on this topic.

### 2.4. The Data Analysis

The primary outcome of this study was the caries status of the children as reported by the parents and their knowledge of the influence of breastfeeding and feeding habits on their child’s oral health. Continuous variables were presented as means (standard deviations), while categorical variables were expressed as numbers and percentages. The normality of continuous variables was tested using the Shapiro–Wilk test. The chi-square test and Fisher’s exact test were used to compare categorical variables between children with and without reported caries, while the Mann–Whitney U test was applied to continuous variables. A multivariable logistic regression model was applied to assess the differences in parental knowledge based on their sociodemographic characteristics and breastfeeding practices, with the results expressed as regression coefficients (beta) and 95% confidence intervals (CIs). Data were analyzed using SPSS version 26.0 for Windows (IBM Corp., Armonk, NY, USA), with the statistical significance set at *p* < 0.05.

## 3. Results

Table 1 presents the sociodemographic data of the parents. A total of 595 parents of children aged 3 to 6 years participated in this study. The mean age of the parents was 35.6 ± 5.1 years (range: 22 to 49). The average number of children per parent in this study was 1.9 ± 1.1, ranging from 0 to 9. The children’s average body mass index (BMI) was 15.3 ± 2.2. Of 559 parents, 33.6% (N = 200) reported having experienced tooth decay among their children. These respondents had a knowledge score of 4.6 ± 2.6 (minimum 0, maximum 11). Additionally, respondents from rural areas exhibited higher caries rates (40.5% vs. 31.1%, *p* = 0.021), as did those from low-income families (72.7%), compared to those from middle- (35.4%) and high-income families (25.1%) (*p* = 0.002). The children of parents with university degrees experienced significantly lower caries rates (30.1%) compared to those whose parents had completed high school (41.1%) or only primary education (100%) (*p* = 0.005). The number of children also influenced caries experience, as parents with three or more children had significantly higher caries rates (42.9%) compared to those with two children (36.1%) or one child (27.2%) (*p* = 0.009). Two-thirds of the parents (N = 359) did not report experiencing tooth decay among their children and demonstrated a knowledge score of 4.9 ± 2.9 (minimum 0, maximum 12).

Table 2 illustrates the data reported by parents about their youngest children. The respondents’ children were nearly evenly distributed by gender, with 50.8% being female and 49.2% male. The majority (66.7%) had an average BMI. Children with a BMI ≤ 14 had a significantly higher caries prevalence (42.5%) compared to those with an average (29.7%) and high (38.5%) BMI (*p* = 0.016).

Table 3 shows the breastfeeding and feeding habits reported in the children. Most of the parents breastfed their children for 13 to 24 months (26.9%), while 13.4% did not breastfeed them at all. One-third of the parents (33.6%) reported breastfeeding or bottle-feeding their children three or more times during the night upon the child’s request. Over 90% stated that water or milk was the beverage most consumed by their children (93.1%). Almost all of the parents reported sharing eating utensils with their children (93.3%, N = 555), which was significantly associated with higher caries rates (*p* = 0.047). Other parameters, such as breastfeeding duration, bottle feeding, and night feeding, were not significantly associated with the occurrence of caries.

Table 4 summarizes the frequency of questions being correctly answered regarding the knowledge of the association between breastfeeding, nutrition, and dental caries. The average knowledge score was 4.8 ± 2.8 out of a possible 12 points, reflecting a low to moderate level of knowledge. Parents provided the highest number of correct answers to the questions regarding the harmful effects on oral health of pacifier use (93.9%), the prolonged consumption of sweetened beverages (85.7%), and nighttime milk feeding as a risk factor for caries development (85.0%). Only 11.1% of the parents recognized the role of extended breastfeeding on a child’s demand in promoting the development of tooth decay. The fewest correct answers were given to the question related to the preventive role of breastfeeding in snoring (7.9%).

Table 5 summarizes the reported daily dietary habits of the respondents’ children. According to the parental reports, most of their children consume fruits (92.6%), meat and fish (87.2%), and vegetables (87.2%) daily. Approximately half of the children consume sweets (55.5%), white bread (56.1%), and white rice or pasta (50.9%). Nearly all of the children drink water every day (98.8%). Other cariogenic foods were consumed less frequently, including cakes (4.4%), snacks (28.1%), chocolate (29.9%), and sweetened beverages (13.3%) daily.

Table 6 illustrates the results of the analysis using a generalized linear model identifying the predictors of a higher total score in the association between breastfeeding, nutrition, and oral health knowledge. Higher education, employment in the healthcare field, breastfeeding children for more than one year, and nighttime breastfeeding more than twice were significantly associated with higher levels of knowledge (*p* < 0.05). Conversely, an average or above average socioeconomic status was associated with lower knowledge scores (*p* < 0.05).

## 4. Discussion

Early childhood caries (ECC) is a multifactorial public health problem influenced by diet, oral hygiene, genetics, socioeconomic factors, and parental knowledge, with prolonged nighttime breastfeeding and frequent sugar consumption increasing the risk by promoting the growth of cariogenic bacteria and demineralization of the tooth enamel [1,2,12,13,14,15,16,17]. The aim of this study was therefore to investigate the breastfeeding and dietary habits of Croatian parents and to assess their knowledge of the impact of these factors on children’s orofacial health. It also aimed to investigate how dietary habits and breastfeeding practices influence the occurrence of early childhood caries. In this study, one-third of the respondents (33.6%, N = 200) reported the presence of caries in their children. This prevalence is roughly in line with the European average of 36% reported in a systematic review from 2024 [3]. Interestingly, the caries rates tended to be higher in rural areas and low-income families in this study (*p* < 0.05). These findings are consistent with the systematic review, which highlights that children from low-income families and rural areas experience a higher prevalence of dental caries due to factors such as limited access to dental care, lower parental education levels, and inadequate oral hygiene practices [27].

Similarly, a 2019 study conducted among school children in Croatia reported that half of the children examined (50.0%) had dental caries, with a prevalence of 46.0% in urban areas and 70.2% in rural areas [28]. A study conducted among Croatian children in Primorsko-Goranska County reported a high prevalence of dental caries in the primary teeth, with three-quarters of 6-year-old children having an average of four primary teeth affected in contrast to only 12% of children in the same age group experiencing caries in their permanent teeth. Furthermore, this study reported a higher prevalence of dental caries among boys compared to that in girls [29]. According to data from the Croatian Central Health Information System for the period 2013–2015, the DMFT index for 6-year-old children was reported to be 4.14, with the values ranging from 3.10 to 5.77. These findings highlight a concerning level of dental caries among young children in Croatia, emphasizing the need for targeted preventive measures and improved oral health education from an early age [5].

The World Health Organization and the American Academy of Pediatrics emphasize that breastfeeding is essential for infant health and recommend exclusive breastfeeding for six months and continued breastfeeding with complementary foods for at least two years [6,7]. In this study, the largest proportion of the respondents (26.9%) breastfed their children for one to two years, whereas 13.4% did not breastfeed them at all. Alarmingly, 42.4% of the parents fed their child on demand once per night, and 24.0% did so twice, while a staggering 33.6% fed their child three or more times during the night. Furthermore, the duration of breastfeeding and night feeding in this study was not associated with a higher prevalence of caries in children. A survey conducted among preschoolers aged 3–5 years in Xiangyun, China, revealed an association between breastfeeding and early childhood caries. Among the participants, 43.8% of the children were breastfed within the first six months and exhibited a slightly lower prevalence of ECC (72.0%) compared to that in the 56.2% of children who received mixed or bottle feeding. However, this difference was not statistically significant [16]. A population-based birth cohort study in southern Brazil investigated whether the duration of breastfeeding was a risk factor for dental caries in the primary dentition, independently of sugar consumption. Its findings indicated that children breastfed for 24 months or longer had a higher number of decayed, missing, and filled primary tooth surfaces (dmfs) and a significantly increased risk of severe early childhood caries (S-ECC) compared to these values in those breastfed for up to 12 months [30]. Similarly, a retrospective longitudinal study assessed the association between long-term breastfeeding and dental caries in children during their third year of life. Said study found that children breastfed for 24 months or more were more likely to have dental caries compared to those breastfed for less than 6 months. Additionally, a higher frequency of sucrose intake and the presence of dental plaque were also associated with an increased risk of dental caries [31]. Conversely, a longitudinal study in Thailand examined the impact of breastfeeding with or without formula milk on dental caries. This study found that full breastfeeding for 6 to 17 months decreased the risk of dental caries in 3-year-old children compared to full breastfeeding for less than 6 months. However, when any breastfeeding (including formula milk) continued for 18 months or more, the caries prevalence increased [32]. The impact of prolonged breastfeeding on early childhood caries remains unclear and multifaceted. While breastfeeding is widely recognized for its protective benefits, some studies have failed to demonstrate a clear association between prolonged breastfeeding and increased caries risk. Although certain studies suggest that frequent nighttime feeding beyond the first year, especially when combined with inadequate oral hygiene, may elevate the risk of caries, other research does not support this claim. The potential risk associated with prolonged exposure to milk lactose during night feeding and its role in promoting cariogenic bacteria remains inconclusive, with mixed findings across the literature [12,13,14].

The transmission of pathogenic bacteria through sharing eating utensils or tasting a child’s food has been identified as a contributing factor to the development of dental caries [18]. In the present study, 93.3% of the parents reported sharing utensils with their children during feeding, demonstrating a statistically significant correlation with an increased occurrence of caries. Similar findings have been reported in other investigations assessing parental knowledge, including a study in which 60.5% of breastfeeding mothers in Israel engaged in the same practice [13]. Furthermore, a survey conducted in the Western Region of Saudi Arabia revealed that 68.0% of the parents were unaware that tooth decay could be caused by bacterial transmission through shared feeding utensils [19]. Several studies have investigated the association between sharing eating utensils and the occurrence of dental caries in children. For instance, research has shown that 14% of mothers reported sharing a spoon with their child, and such behavior was linked to an increased risk of caries transmission [33]. Additionally, a study among children aged 0–5 years found that a significant factor in developing dental cavities was shared food between a caregiver/parent and their child, highlighting the potential role of bacterial transmission in caries development [34]. These findings underscore the role of intra-familial bacterial transmission in the development of dental caries and emphasize the need for increased awareness of its impact on children’s oral health.

Breastfeeding plays a crucial role in orofacial development by facilitating physiological sucking patterns that contribute to the optimal jaw growth and dental arch formation, potentially lowering the incidence of malocclusion [8,9,10]. However, the association between prolonged breastfeeding and early childhood caries (ECC) remains a subject of ongoing investigation. While breastfeeding is generally regarded to be protective against dental caries, evidence suggests that frequent nocturnal feeding beyond the first year of life, particularly in the absence of adequate oral hygiene, may predispose children to ECC. This increased susceptibility is attributed to prolonged intraoral exposure to lactose, which, under the conditions of a reduced salivary flow during sleep, may promote an environment conducive to the proliferation of cariogenic microorganisms, ultimately enhancing the risk of enamel demineralization and caries development [12,13,14]. In this study, parents did not demonstrate satisfactory levels of knowledge and awareness regarding the effects of breastfeeding on their children’s oral health. Only 11.1% of the parents acknowledged the potential role of prolonged, demand-driven breastfeeding beyond one year in increasing the risk of tooth decay. Furthermore, only 7.9% recognized breastfeeding’s protective effect against snoring. In other studies, similar trends were observed. Research among mothers in Tel Aviv found that only 47.8% of them recognized nocturnal breastfeeding as a risk factor for dental caries [13], while a study in Saudi Arabia reported that 53.1% of parents acknowledged this association [19]. Studies conducted in Croatia have reported concerning findings regarding parental awareness of the relationship between breastfeeding and dental caries. A 2022 study indicated that 90.77% of pregnant women did not recognize breastfeeding as a potential risk factor for caries development [35]. The most alarming results were observed in a study among pregnant women in Rijeka, Croatia, where none of the participants demonstrated awareness of this association [24]. These findings underscore a critical lack of awareness among parents, particularly in Croatia, about the potential relationship between breastfeeding and the development of dental caries. This highlights the need for enhanced public health education to address this knowledge gap and promote better oral health practices.

It is crucial to highlight the significant role of nutrition in the development of early childhood caries. Diets high in sugary snacks, sweetened beverages, and processed foods contribute to the onset of dental caries by fostering an environment conducive to enamel demineralization and cariogenic bacterial growth [15,16,17]. Conversely, a well-balanced diet, rich in calcium, phosphorus, vitamin D, and other essential nutrients, plays a vital role in enamel remineralization and the maintenance of optimal oral health. Parental promotion of healthy eating habits, including the regular consumption of fruits, vegetables, dairy products, meat, and fish, supports enamel integrity and reduces the risk of caries formation [19,20]. In this study, most of the children had healthy dietary habits, including daily consumption of fruits (92.6%), meat and fish (87.2%), and vegetables (87.2%). Only 13.3% of the parents reported that their children consumed sweetened beverages on a daily basis. A study among Chinese preschoolers revealed that 33.2% of children consumed desserts at least once a day and yet exhibited a similar prevalence of early childhood caries (74.6%) to that in children who ate desserts occasionally or never (74.2%). Additionally, 9.8% of the children consumed sweet drinks, and 25.5% ate candies or chocolates more than once a day. However, these factors were not statistically significant in terms of a higher prevalence of caries [16]. Furthermore, a study conducted among 3–5-year-old preschool children in Kisarawe, Tanzania, found a significant positive association between the dmft score index and total sugar exposure [17]. A study investigating the dietary habits among Egyptian schoolchildren also found a significant positive correlation between dmft scores and factors such as BMI and the consumption of legumes, sweetened milk and juice, soft drinks, and desserts in terms of the effects on the primary dentition [18]. Therefore, it can be concluded that excessive sugar intake during childhood is a major risk factor for poor oral health, significantly contributing to the development of dental caries and other related oral issues. The consistently high consumption of sugary foods and beverages not only increases the likelihood of caries but also fosters an environment conducive to the growth of cariogenic bacteria, which accelerates tooth decay. This pattern of unhealthy dietary habits, if sustained over time, can have long-term consequences for both the immediate and future oral health of children, underscoring the importance of early dietary interventions and preventive measures to mitigate these risks [36].

This study has several limitations that need to be considered. Firstly, the sample size is relatively small, which may restrict the generalizability of the findings to the broader population of parents with children aged 3 to 6 years. Secondly, since this study employed online data collection, it relied on volunteer sampling rather than probability sampling, which limits the ability to generalize its findings to the broader population. This study also only included parents who had access to the internet and actively sought information on social media platforms where the survey was shared. This inherently limited the sample to individuals who were more likely to be internet-savvy and engaged in online communities, which may not have represented the entire population of parents. Additionally, another limitation is the higher proportion of female respondents in this study, which may have introduced a gender bias. This overrepresentation of female participants may affect the sample’s representativeness and may not fully reflect the perspectives and behaviors of both parents. Future studies should consider alternative, more diverse recruitment strategies to address these biases and improve the generalizability of the findings. Another limitation is the lack of adjustments for potential confounders in the multivariable logistic regression model. This is a significant limitation, as failing to adjust for important variables may affect the accuracy and reliability of the results. Future studies should incorporate appropriate adjustments to control for potential confounding factors, thereby enhancing the validity and robustness of the findings. Lastly, this study relied on self-reported data rather than clinical examinations, which may have introduced bias and limited the accuracy of the findings. Future studies should aim to include larger and more diverse samples to improve the generalizability of the findings. Additionally, they should consider using probability sampling techniques to reduce potential sampling bias. To enhance the reliability of the data, future research could incorporate clinical examinations alongside self-reported data. Longitudinal studies would also be valuable for exploring the long-term effects of dietary habits on oral health. Moreover, future investigations could include both male and female participants in more balanced proportions to avoid gender bias and ensure more representative results. Lastly, this study can serve as a valuable tool for assessing parents’ knowledge regarding the impact of breastfeeding and nutrition on oral health. The findings highlight significant gaps in awareness, emphasizing the need for targeted interventions to improve parental understanding. Increasing awareness of the importance of breastfeeding practices and healthy dietary habits can play a key role in preventing early childhood caries. Public health campaigns and educational programs should focus on providing parents with evidence-based information to enhance their ability to make informed decisions that support their children’s oral health.

## 5. Conclusions

This study reveals a significant gap in parents’ knowledge regarding the impact of breastfeeding and nutrition on oral health, particularly concerning early childhood caries. Despite many parents reporting healthy dietary habits in their children, there is a noticeable lack of awareness about the risks associated with prolonged nocturnal breastfeeding and excessive sugar consumption. These findings emphasize the need for targeted educational interventions to improve parental understanding and raise awareness about the importance of proper breastfeeding practices, balanced nutrition, and oral hygiene in preventing dental caries.

## Figures and Tables

**Table 1:** Sociodemographic data of respondents (parents).

Characteristic		Total (N = 595)	Tooth Decay Experience		*p*-Value
			Yes (N = 200)	No (N = 395)	
**Gender**	Female	562 (94.5)	182 (91.0)	380 (96.2)	0.013 *
	Male	33 (5.5)	18 (9.0)	15 (3.8)	
**Age**	≤29	74 (12.4)	24 (12.0)	50 (12.7)	0.895
	30–39	388 (65.2)	133 (66.5)	255 (64.6)	
	≥40	133 (22.4)	43 (21.5)	90 (22.8)	
**Education level**	Elementary school	2 (0.3)	2 (1.0)	0 (0)	0.005 *
	High school	175 (29.4)	72 (36.0)	103 (26.1)	
	University	418 (70.3)	126 (63.0)	292 (73.9)	
**Employment status**	Unemployed	69 (11.6)	29 (14.5)	40 (10.1)	0.077
	Employed	526 (88.4)	171 (85.5)	355 (89.9)	
**Employment in the healthcare field**	No	487 (81.8)	167 (83.5)	230 (81.0)	0.266
	Yes	108 (18.2)	33 (16.5)	75 (19.0)	
**Socioeconomic status**	Below average	11 (1.8)	8 (4.0)	3 (0.8)	0.002 *
	Average	437 (73.4)	155 (77.5)	282 (71.4)	
	Above average	147 (24.7)	37 (18.5)	110 (27.8)	
**Place of living**	Rural	158 (26.6)	64 (22.0)	94 (23.8)	0.021 *
	Urban	437 (73.4)	136 (68.0)	301 (76.2)	
**Number of children**	1	253 (42.5)	69 (34.5)	184 (46.6)	0.009 *
	2	230 (38.7)	83 (41.5)	147 (37.2)	
	≥3	112 (18.8)	48 (24.0)	64 (16.2)	

Data are presented as the frequency (percentages). χ^2^—chi-square test or Fisher’s exact test, * *p* < 0.05.

**Table 2:** Characteristics of the respondents’ youngest preschool-aged children.

Characteristic		Total (N = 595)	Tooth Decay Experience		*p*-Value
			Yes (N = 200)	No (N = 395)	
**Gender**	Male	293 (49.2)	106 (53.0)	187 (47.3)	0.112
	Female	302 (50.8)	94 (47.0)	208 (52.0)	
**Age**	3	191 (32.1)	36 (13.0)	165 (41.8)	≤0.001 *
	4	163 (27.4)	52 (26.0)	111 (28.1)	
	5	100 (16.8)	46 (23.0)	54 (13.7)	
	6	141 (23.7)	76 (38.0)	65 (16.5)	
**BMI**	≤14	141 (23.7)	60 (30.0)	81 (20.5)	0.016 *
	15–17	397 (66.7)	118 (59.0)	279 (70.6)	
	≥18	57 (7.6)	22 (11.0)	35 (8.9)	

Data are presented as the frequency (percentages). χ^2^—chi-square test or Fisher’s exact test, * *p* < 0.05.

**Table 3:** Information related to the breastfeeding and other nutritional habits of the respondents’ children.

Characteristic		Total (N = 595)	Tooth Decay Experience		*p*-Value
			Yes (N = 200)	No (N = 395)	
**Duration of breastfeeding**	Not breastfed	80 (13.4)	22 (11.0)	58 (14.7)	0.536
	≤6 months	131 (22.0)	51 (25.5)	80 (20.3)	
	7–12 months	118 (19.8)	38 (19.0)	80 (20.3)	
	13–24 months	160 (26.9)	54 (27.0)	106 (26.5)	
	>24 months	106 (17.8)	35 (17.5)	71 (18.0)	
**Duration of bottle feeding**	Not bottle-fed	282 (47.4)	96 (48.0)	186 (47.1)	0.247
	1 year	198 (33.3)	59 (29.5)	139 (35.2)	
	2 years	81 (13.6)	29 (14.5)	52 (13.2)	
	≥3 years	34 (5.7)	16 (8.0)	18 (4.6)	
**Frequency of nighttime breastfeeding/bottle feeding**	Once	252 (42.4)	77(38.5)	175 (44.3)	0.348
	Twice	143 (24.0)	49 (24.5)	94 (23.8)	
	≥Three times	200 (33.6)	74 (37.0)	126 (31.9)	
**Most frequently consumed beverage**	Water/milk	554 (93.1)	176 (88.8)	378 (95.7)	≤0.001 *
	Sugary drinks	41 (6.9)	24 (12.0)	17 (4.3)	
**Age at the introduction of complementary feeding (solid foods)**	4 months	107 (18.0)	37 (18.5)	70 (17.7)	0.638
	6 months	437 (73.4)	143 (71.5)	294 (74.4)	
	>6 months	51 (8.6)	20 (10.0)	31 (7.8)	
**Sharing eating utensils with parents**	Yes	555 (93.3)	191 (95.5)	364 (92.2)	0.047 *
	No	40 (6.7)	9 (4.5)	31 (7.8)	

Data are presented as the frequency (percentages). χ^2^—chi-square test or Fisher’s exact test, * *p* < 0.05.

**Table 4:** Parental knowledge assessment of the association between breastfeeding and dietary habits and specific oral conditions.

Question	Total (N = 595)	Tooth Decay Experience		*p*-Value
		Yes (N = 200)	No (N = 395)	
**Breastfeeding prevents oral candidiasis (“Yes”)**	144 (24.2)	53 (26.5)	91 (23.0)	0.203
**Breastfeeding prevents snoring (“Yes”)**	47 (7.9)	8 (4.0)	39 (9.9)	0.007 *
**Breastfeeding prevents atypical swallowing patterns (“Yes”)**	132 (22.2)	33 (16.5)	99 (29.1)	0.011 *
**Breastfeeding prevents malocclusion**	113 (19.0)	29 (14.5)	84 (21.3)	0.029 *
**Breastfeeding enables proper chewing function (“Yes”)**	188 (31.6)	62 (31.0)	126 (31.9)	0.450
**Breastfeeding enables proper facial growth and development (“Yes”)**	183 (30.8)	56 (28.0)	127 (32.2)	0.173
**Breastfeeding enables proper teeth and gum development (“Yes”)**	220 (36.9)	61 (30.5)	159 (40.3)	0.012 *
**Extended breastfeeding on a child’s demand beyond one year promotes the development of tooth decay (“Yes”)**	66 (11.1)	26 (13.0)	40 (10.1)	0.179
**Frequent bottle feeding with milk leads to the development of early childhood caries (“Yes”)**	506 (85.0)	174 (87.0)	332 (84.1)	0.204
**Frequent and prolonged bottle feeding with sweetened instant or natural chamomile tea, and sweetened store-bought or fresh fruit juices, harms teeth (“Yes”)**	510 (85.7)	170 (85.0)	340 (86.1)	0.405
**Frequent use of a pacifier sweetened with sugar, honey, or juice, especially at night, harms teeth (“Yes”)**	559 (93.9)	190 (95.0)	369 (93.4)	0.284
**A mother’s diet during pregnancy affects the child’s teeth (“Yes”)**	240 (40.3)	74 (37.0)	166 (42.0)	0.137

Data are presented as numbers (percentages). χ^2^—chi-square test or Fisher’s exact test, * *p* < 0.05.

**Table 5:** Frequency of daily dietary habits of the respondents’ children.

Foods or Drinks	Total (N = 595)	Tooth Decay Experience		*p*-Value
		Yes (N = 200)	No (N = 395)	
**Sweets (“Yes”)**	330 (55.5)	126 (63.0)	204 (51.6)	0.005 *
**Cakes (“Yes”)**	26 (4.4)	18 (9.0)	8 (2.0)	≤0.001 *
**Snacks (“Yes”)**	167 (28.1)	73 (36.5)	94 (23.8)	≤0.001 *
**Chocolate (“Yes”)**	178 (29.9)	70 (35.0)	108 (27.3)	0.034 *
**Natural juice (“Yes”)**	146 (24.5)	64 (32.0)	82 (20.8)	0.002 *
**Sugar-coated cereal (“Yes”)**	96 (16.1)	39 (19.5)	57 (14.4)	0.072
**Sweetened beverages (“Yes”)**	79 (13.3)	37 (18.5)	42 (10.6)	0.006 *
**Milk (“Yes”)**	402 (67.6)	132 (66.0)	270 (68.4)	0.312
**Water (“Yes”)**	588 (98.8)	197 (98.5)	391 (99.0)	0.436
**Yogurt and cheese (“Yes”)**	393 (66.1)	130 (65.0)	163 (66.6)	0.384
**Fruit (“Yes”)**	551 (92.6)	187 (93.5)	364 (92.2)	0.339
**Vegetables (“Yes”)**	531 (89.2)	173 (86.5)	358 (90.6)	0.083
**Meat and fish (“Yes”)**	519 (87.2)	169 (84.5)	350 (88.6)	0.100
**White bread (“Yes”)**	334 (56.1)	120 (60.0)	214 (54.2)	0.103
**Rice and pasta (“Yes”)**	303 (50.9)	106 (53.0)	197 (49.9)	0.263

Data are presented as numbers (percentages). χ^2^—chi-square test or Fisher’s exact test, * *p* < 0.05.

**Table 6:** Generalized linear model analysis of predictors for higher breastfeeding, nutrition, and oral health knowledge score.

Characteristic		β (95% CI)	*p*-Value
**Gender**	Female	Reference	
	Male	−0.892 (−1.817–0.032)	0.058
**Age**	≤29	Reference	
	30–39	−0.262 (−0.943–0.419)	0.450
	≥40	−0.050 (−0.846–0.746)	0.902
**Education level**	Elementary school	Reference	
	High school diploma	3.890 (0.194–7.586)	0.039 *
	University education	4.467 (0.761–8.174)	0.018 *
**Employment status**	Unemployed	Reference	
	Employed	−0.194 (−0.879–0.492)	0.580
**Employment in the healthcare field**	No	Reference	
	Yes	2.109 (1.557–2.661)	≤0.001 *
**Socioeconomic status**	Below average	Reference	
	Average	−2.945 (−4.049–−0.821)	0.003 *
	Above average	−2.054 (−3.731–−0.377)	0.016 *
**Place of living**	Rural	Reference	
	Urban	−0.054 (−0.543–0.436)	0.829
**Number of children**	1	Reference	
	2	−0.031 (−0.500–0.438)	0.897
	≥3	−0.075 (−0.633–0.063)	0.801
**Duration of breastfeeding**	Not breastfed	Reference	
	≤6 months	0.649 (−0.103–1.401)	0.091
	7–12 months	0.580 (0.255–1.415)	0.133
	13–24 months	1.203 (0.215–2.119)	0.017 *
	>24 months	1.306 (0.205–2.408)	0.020 *
**Duration of bottle feeding**	Not bottle-fed	Reference	
	1 year	0.337 (−0.451–1.125)	0.402
	2 years	0.326 (−0.606–1.257)	0.493
	≥3 years	0.365 (−0.799–1.529)	0.539
**Frequency of nighttime breastfeeding/bottle feeding**	Once	Reference	
	Twice	0.744 (0.170–1.318)	0.011 *
	≥Three times	0.743 (0.146–1.340)	0.015 *
**Most frequently consumed beverage**	Water/milk	Reference	
	Sugary drinks	−0.369 (−1.237–0.500)	0.405
**Age at introduction of complementary feeding (solid foods)**	4 months	Reference	
	6 months	−0.141 (−0.751–0.470)	0.652
	>6 months	0.091 (−0.862–1.045)	0.851
**Sharing eating utensils with parents**	Yes	−0.863 (−1.739–0.013)	0.053
	No	Reference	
**Experience of tooth decay**	Yes	−0.322 (−0.788–0.143)	0.175
	No	Reference	

Data are presented as numbers. The reference knowledge category is “low”. β, correlation coefficient; 95% CI, 95% confidence interval. * *p* ≤ 0.05.

## Data Availability

The data are available from the corresponding author upon reasonable request.
